# Means and Long-Term Trends of Global Coastal Zone Precipitation

**DOI:** 10.1038/s41598-019-41878-8

**Published:** 2019-04-01

**Authors:** Scott Curtis

**Affiliations:** Distinguished Professor in Natural Sciences and Mathematics, Department of Geography, Planning, and Environment, East Carolina University, Greenville, NC 27858 USA

## Abstract

Precipitation in the coastal zone is important to the socio-economic and ecological well-being of the world. Meteorologically, precipitation is generated by unique mechanisms at the land-sea interface, which is why coastal zone precipitation is not well resolved by global climate models. Yet, to date, much more effort has been placed in analyzing global precipitation over the oceans and land. In this study, global coastal zone precipitation is quantified by selecting Global Precipitation Climatology Centre V2018 0.5° grid cells in 50 km zones from the shoreline into the interior. The transition from maritime to continental precipitation regimes is revealed in the long-term (1931–2010) average, as there is a pronounced coast-to-interior decline in rainfall from approximately 911.5 mm yr^−1^ within 50 km of the coast to 727.2 mm yr^−1^ from 100 to 150 km away from the coast. Globally, coastal zone precipitation peaks in boreal summer, extending into fall for precipitation at the coastline. Dividing the long-term record into early and late 40-year periods reveals an increasing trend in precipitation in the coastal zone, with the interior increasing faster than at the coastline. Averaging over 30-year climate normals from 1931–60 to 1981–2010 further confirms this result. A seasonal analysis reveals that the upward trends, and discrepancy between the coast and inland are maximized in the austral summer season. Interestingly, from May to September there is a declining trend in rainfall at the coastline, whereas the interior only shows minimal declines in August and September. Potential forcing mechanisms that could favor a wetter interior coastal zone include changes in the sea breeze circulation, urban heat island effect, or precipitation content associated with synoptic systems or monsoonal circulations.

## Introduction

Coastal environments across the globe are threatened by sea level rise due to warming seas and melting glaciers, but an often-overlooked threat is changes in macroclimatic features, for example precipitation, which will impact coastal ecosystem services and economies^1,2^. Many studies have examined regional climates of coastal precipitation and their variability (summarized below), but a quantification of global-scale coastal precipitation is lacking. Furthermore, even fewer studies have looked at the transition of mean precipitation from the coast to the interior. However, it is expected that coastal areas with uniform physiographic features would experience a gradient in rainfall where the highest amounts would be found at the land-sea interface due to the ready supply of heat and moisture. It should be noted that not all coastal regions behave in this manner, especially coasts where the orientation of mountain chains lead to orographically enhanced rainfall.

Consistent with this picture, global estimates of oceanic precipitation are consistently higher than terrestrial precipitation. The annual average land precipitation, excluding Antarctica, from 1951–2000 is estimated at 850 mm by the Global Precipitation Climatology Centre (GPCC) 0.5° latitude/longitude gridded product^3^. Another recent study by the GPCC increased this total to 854.7 mm yr^−1^ after additional gauges were added and updates were made to the gauge undercatch algorithm^4^. These authors also found that there was no significant trend in land surface precipitation when examining climate normals from 1931–60 to 1981–2010^4^. Another study, which investigated annual GPCC precipitation from 1901 to 2008, along with estimates from the Climate Research Unit (CRU), and the University of Delaware (all at 0.5° resolution), found similar terrestrial climatologies of 716 mm (a gauge undercatch correction was not applied)^5^. They also found trends at various periods in the record ending with an upward trend since 1992^5^.

With regards to ocean precipitation, the Global Precipitation Climatology Project (GPCP) version 2.3 annual rainfall estimate is 1065.8 mm yr^−1^ (2.89 mm day^−1^)^6^, which is very close to a water vapor modeled estimate of 1069 mm yr^−1^ ^7^. Thus, coastal precipitation is likely in between this range of values. In fact, GPCP version 2.1 overestimated terrestrial precipitation as 923 mm yr^−1^, because the authors classified grid boxes as terrestrial if any portion (however small) contained land, thus introducing a “maritime bias”^3^.

Much of the previous coastal climate research has been regional in nature, with a few notable exceptions. Recently, a study quantified precipitation as a function of distance from the coast in the tropics with Tropical Rainfall Measuring Mission data^8^. They found that precipitation peaks at the coastline and then drops sharply off seaward and landward, with the steepest decline being on the land side^8^. Another study used pattern recognition to isolate global coastal precipitation related to land-sea interactions from a satellite product^9^. The authors found that the Maritime Continent, Bight of Panama, Madagascar, and the Mediterranean are regions where 40–60% of the total rainfall is generated by coastline effects^9^. Neither study considered long-term trends. Many coastal regions around the world experience significant precipitation and have been extensively studied^10–23^. A few examples are given in the following.

In many cases in the tropics, near shore orography can lead to extreme precipitation at the coast. The diurnal cycle of heating combined with elevated terrain leads to added lift and mesoscale convective systems crossing the coastline^10–13^. Mountains also block and slow monsoonal flow at the western coasts of India, Myanmar, Thailand and the Philippines leading to intense precipitation at the coasts^14^. Globally, tropical cyclones (TCs) contribute from 5 to 10% of the annual terrestrial precipitation, but the contributions decrease sharply within 150 km of the coast^15^. However, TCs can play a larger role regionally. For example, 15–20% of coastal precipitation in the Southeast US is attributed to TCs^16^, while numbers reach 20–40% on the west coast of Australia^17^. Extreme precipitation in the winter season accounts for 60% of precipitation in coastal Mediterranean cities^18^. Furthermore, much of the Mediterranean coast has experienced drying from 1901 to 2009 except for northern Africa, southern Italy, and the western Iberian Peninsula^19^. Extreme precipitation is increasing over most of the West Coast of North America according to an analysis of stations in the coastal zone of the US and British Columbia^20^. Finally, one of the few studies to examine the relationship between precipitation and proximity to the coast occurred in Greenland^21^. A coastal station in the northwest showed a statistically significant increase in precipitation from 1982 to 2000, but precipitation was largely unchanged during that same period for two stations further inland.

Rather than investigate coastal precipitation regionally, the intent of the current study is to scale-up to the globe and quantify precipitation amounts and long-term changes from the coastline to 150 km inland. This distance is somewhat arbitrary, but has been used to denote the “coastal zone” (CZ). For example, one study reported that 44% of the global population lives within 150 km of large water bodies and 37% within 100 km^24^. Sea breezes, and their associated precipitation fronts, are also known to propagate 150 km inland^9^. Here 50 km bins are used, consistent with previous work^8^. To account for global precipitation across these narrow bands in a consistent manner, a high-resolution gauge-based gridded data set is required. GPCC is very useful for examining precipitation within the CZ, and the specifics of the version 2018 product and coastal precipitation metrics can be found in the Data and Methodology section. However, the primary advantage of GPCC is its large population of rain gauges, especially in the CZ. In fact, for other products, like CRU, coastal rainfall is smoothed inland owing to the sparse gauge network^3^. The outline of the study is as follows: coastal precipitation climatologies and trends are given in section 2, possible mechanisms to help explain the results are proposed in section 3, and conclusions are offered in section 4.

## Results

### Long-term climatology

**Figure 1:**
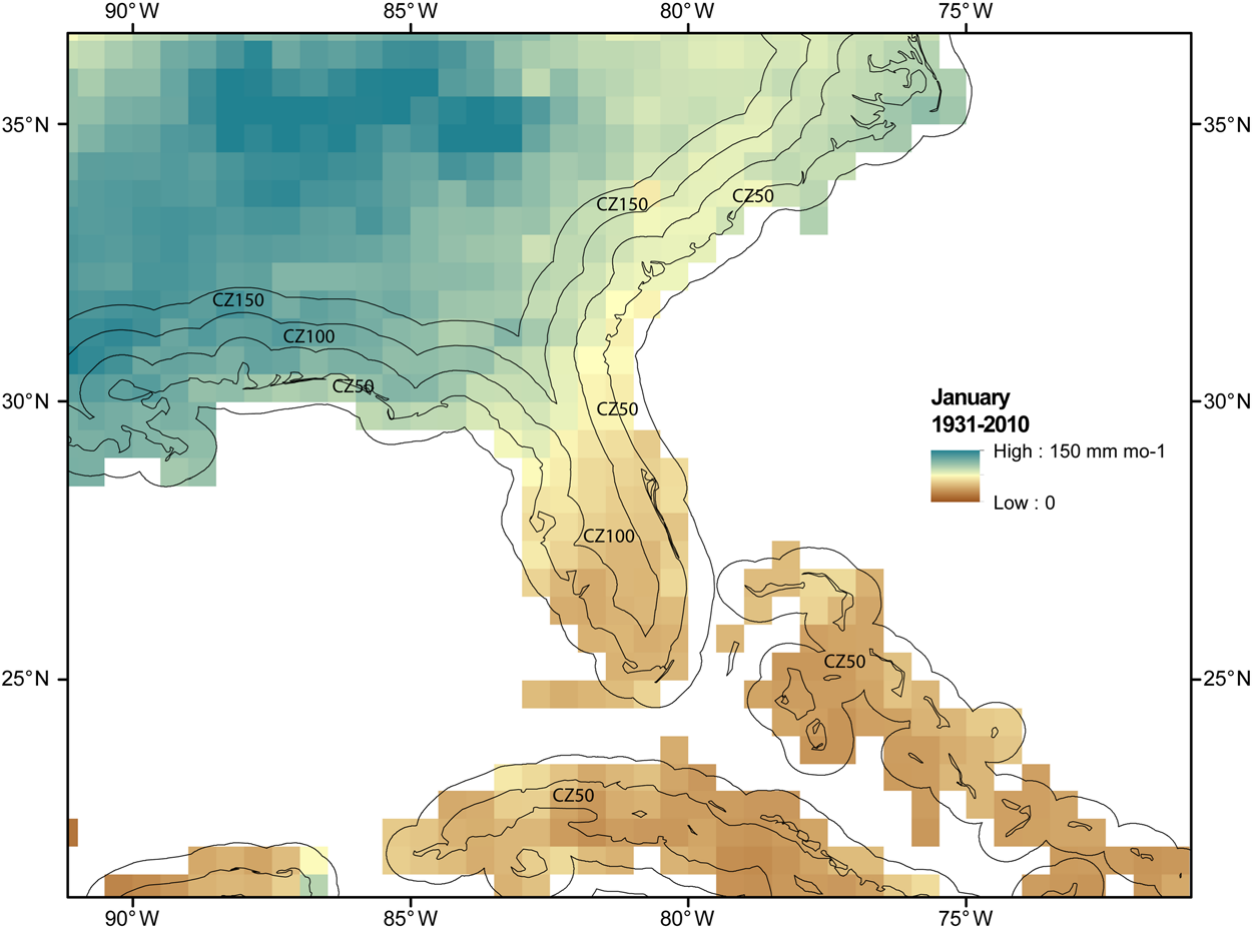
Regional example: January 1931 to 2010 climatology of precipitation in mm mo^−1^ for the Southeast US. Zones within 50 km of the coastline, between 50 km and 100 km of the coastline, and between 100 km and 150 km of the coastline are labeled as CZ50, CZ100, and CZ150 respectively. Map was produced using Desktop ArcMap version 10.5.1 (https://www.esri.com/en-us/home).

Monthly analysis and observation-only data from 1931–2010 was extracted from GPCC using Geographic Information Science (GIS) for three populations: 0.5° grid boxes with center points located within 50 km of the coastline (CZ50), those between 50 and 100 km of the coastline (CZ100), and those between 100 and 150 km of the coastline (CZ150). See Data and Methods section for more information. An example of the analysis product is given in Fig. 1 for the Southeast US. The number of grid boxes in each population and the annual average from 1931 to 2010 is presented in Table 1. The number of CZ50 grid boxes is about 3.5 times that of CZ100, where inlets, bays, peninsulas, and especially islands all account for this discrepancy as indicated in Fig. 1. There are 1000 less grid boxes in CZ150 than CZ100. As expected from the introductory literature review, the largest annual mean is on the coastline (CZ50) and these values decrease inland. CZ50 is within the expected range between land precipitation (~700 mm yr^−1^) and oceanic precipitation (~1070 mm yr^−1^). Progressing inland, values decline towards the overall land average. Interestingly, GPCC shows a rapid reduction in precipitation (−123.8 mm yr^−1^, −14%) from the sea to 100 km inland. From 100 to 150 km the trend continues, but is cut in half (−60.5 mm yr^−1^, −8%). The corresponding numbers of grid boxes for the observation-only product are much less, however the spatial distribution of grid boxes is globally extensive and representative (see Fig. S1). The loss of high latitude coastal precipitation increases the mean rainfall towards the coastal tropical average^8^. The decline in rainfall from CZ50 to CZ150 is even more pronounced: −204.7 mm yr^−1^ (−17%) from CZ50 to CZ100 and −134.4 mm yr^−1^ (−13%) from CZ100 to CZ150, consistent with previous work^8^.

**Table 1:** Number of grid boxes and 1931–2010 mean annual precipitation for CZ50, CZ100, and CZ150 for the GPCC V2018 analysis product and the GPCC V2018 observation-only product.

	CZ50		CZ100		CZ150	
GPCCV2018	N = 17,293	911.5 mm yr^−1^	N = 4,888	787.7 mm yr^−1^	N = 3,873	727.2 mm yr^−1^
GPCCV2018 Obs.	N = 3,701	1209 mm yr^−1^	N = 1,221	1004.3 mm yr^−1^	N = 1,056	869.9 mm yr^−1^

**Figure 2:**
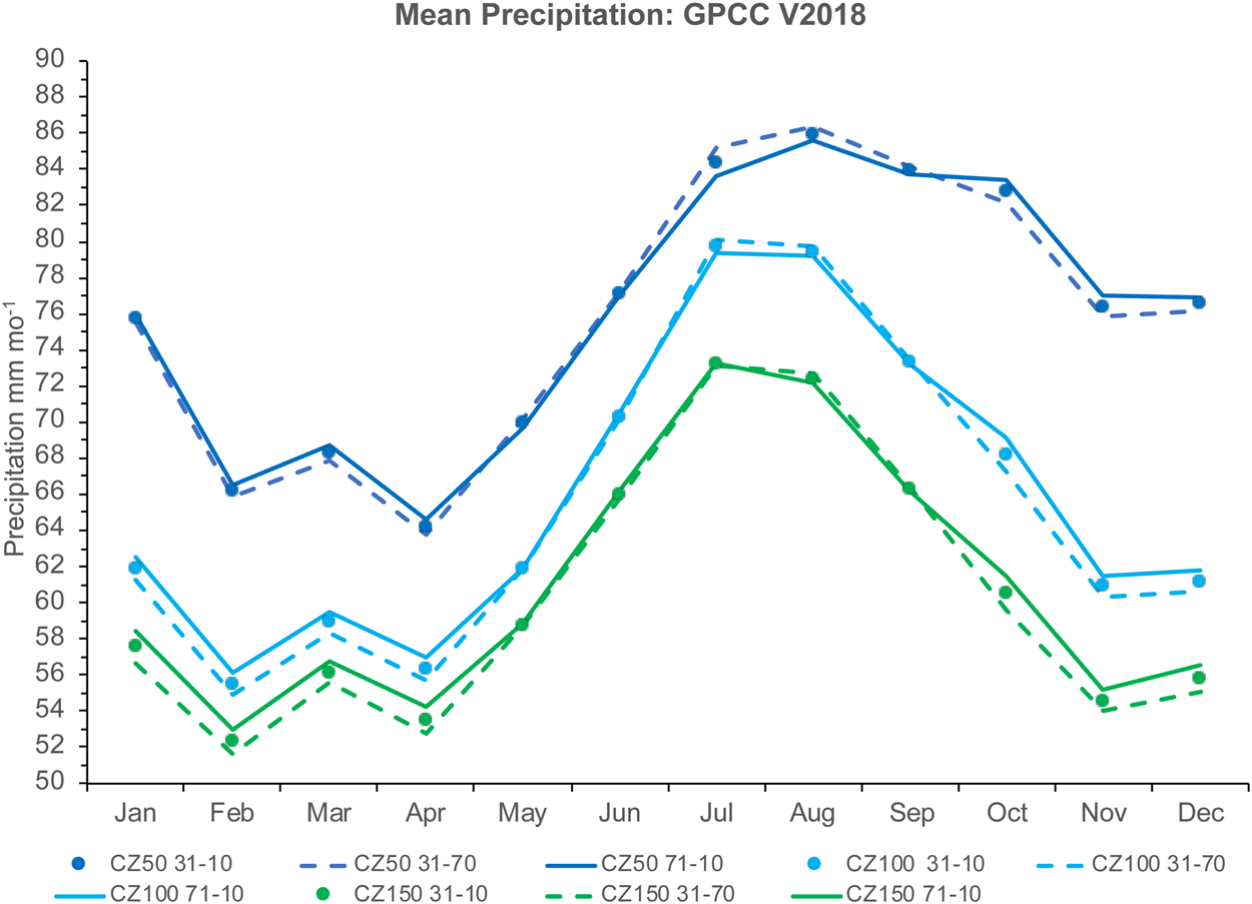
Annual cycle of coastal zone precipitation (mm mo^−1^) for 1931 to 2010 (circles), 1931 to 1970 (dashed lines), and 1971 to 2010 (solid lines). CZ50 is in dark blue, CZ100 in cyan, and CZ150 in green.

Figure 2 shows the annual cycle of the global coastal precipitation measures from the GPCC analysis product. Both CZ100 and CZ150 resemble the annual cycle of terrestrial precipitation as reported in an earlier study^4^. However, the annual cycle of coastline precipitation (CZ50) appears to contain features of previously constructed land and ocean climatologies^25^. CZ50 reaches a maximum in August, which agrees with the GPCP oceanic average, and has much higher values than CZ100 from September to January. This may be related to the climate of the Northern Hemisphere (e.g. monsoon and tropical cyclone season) as the coastline (CZ50) population is mostly located in this hemisphere (82% of the grid boxes).

### Trends: 1931–2010

**Figure 3:**
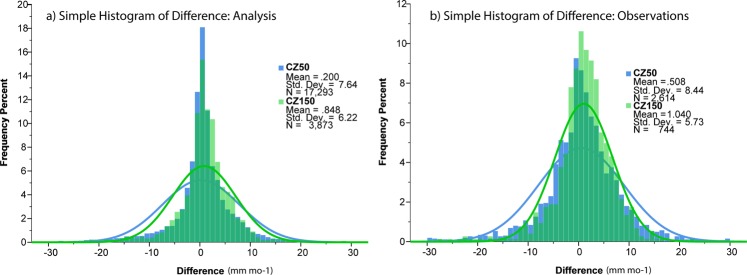
Percentage histograms for all 1971–2010 minus 1931–1970 differences in CZ50 (blue bars) and CZ150 (green bars) in mm mo^−1^. Best fit lines are also drawn for CZ50 (blue line) and CZ150 (green line). (**a**) Is for all analysis grid boxes and (**b**) is for observation grid boxes.

Precipitation averages were also taken from the first half of the record (1931–1970) and the second half (1971–2010) and differences were computed to determine if changes in coastal precipitation had occurred (Table 2). An increase in precipitation is observed in the three coastal domains for both the analysis and observation-only products, but precipitation is changing most rapidly in the interior. For the analysis product, CZ150 has increased by 10.2 mm yr^−1^ or 1.4% of the long-term mean, while CZ50 has only increased by 2.4 mm yr^−1^ or 0.3%. Since the trend is over forty years, CZ150 is increasing at a rate of 0.35% decade^−1^, which is in good agreement with previously computed trends in global precipitation^26^ and theorized trends based on the rate of warming during this time period^27^. Figure 3 shows the histograms of the differences between the 1971–2010 means minus the 1931–1970 means for CZ50 and CZ150 (analysis and observations). Precipitation is given in mm mo^−1^, and percent of total is used for ease of comparison. The distributions are significantly different from each other (p < 0.001), and in both cases CZ50 has a larger standard deviation than CZ150. Generally, the coastal grid boxes have greater frequency of negative differences and the largest positive differences (>+10 mm mo^−1^). Whereas, the interior grid boxes have a greater frequency of precipitation differences from 0 to +10 mm mo^−1^.

**Table 2:** Mean annual precipitation over two time periods: 1931–1970 and 1971–2010 for CZ50, CZ100, and CZ150 of the GPCC V2018 analysis and observation-only products.

	CZ50		CZ100		CZ150	
GPCC V2018	31–70 n = 17,293 910.3 mm yr^−1^	71–10 n = 17,293 912.7 mm yr^−1^	31–70 n = 4,888 783.7 mm yr^−1^	71–10 n = 4,888 791.6 mm yr^−1^	31–70 n = 3,873 722.0 mm yr^−1^	71–10 n = 3,873 732.2 mm yr^−1^
	∆ +2.4 mm yr^−1^ (+0.3%)		∆ +7.9 mm yr^−1^ (+1.0%)		∆ +10.2 mm yr^−1^ (+1.4%)	
GPCC V2018 Obs.	31–70 n = 2,614 1200.8 mm yr^−1^	71–10 n = 2,614 1207.0 mm yr^−1^	31–70 n = 859 1005.2 mm yr^−1^	71–10 n = 859 1012.4 mm yr^−1^	31–70 n = 744 859.1 mm yr^−1^	71–10 n = 744 871.6 mm yr^−1^
	∆ +6.1 mm yr^−1^ (+0.5%)		∆ +7.2 mm yr^−1^ (+0.7%)		∆ +12.5 mm yr^−1^ (+1.4%)	

Absolute differences between the time periods are given as well as the percent differences.

**Figure 4:**
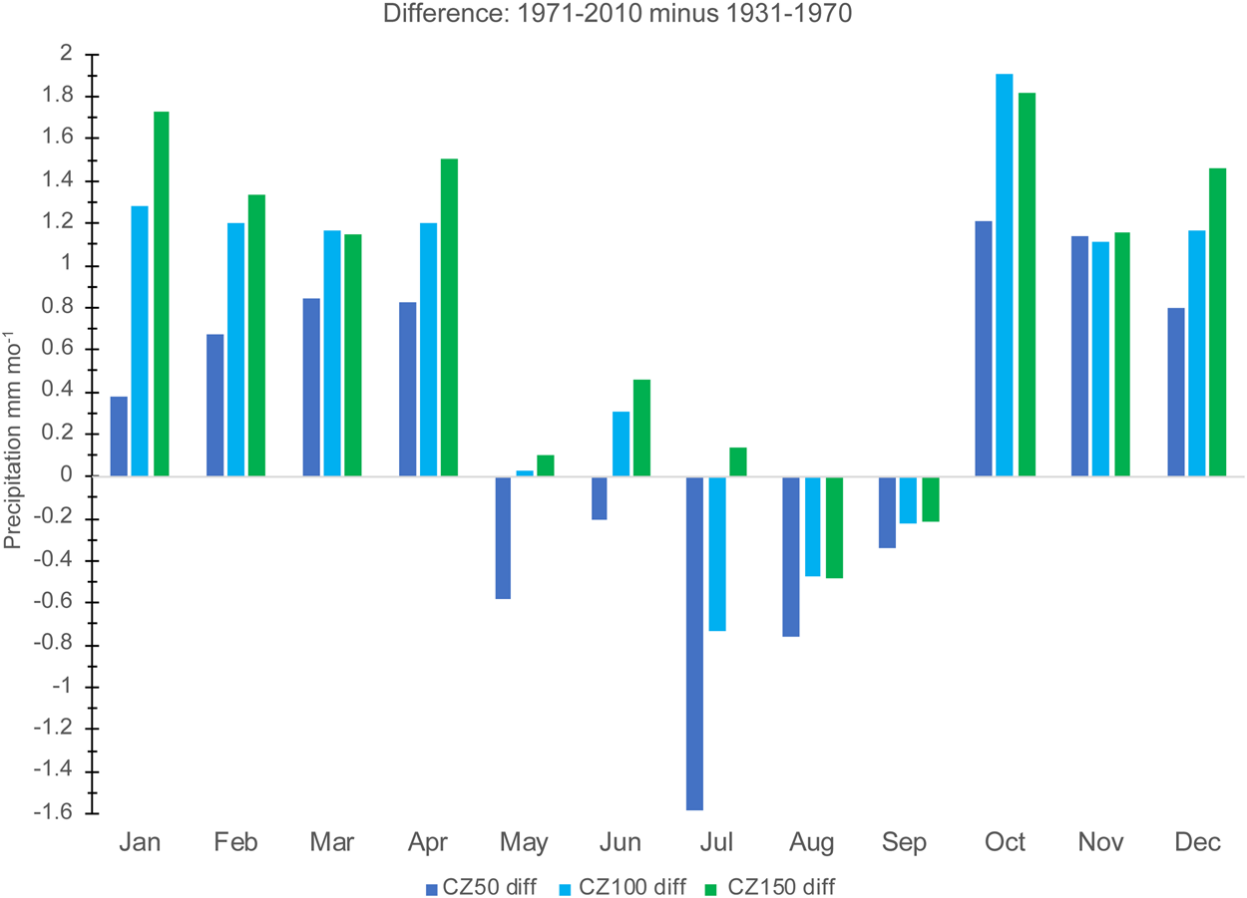
Annual cycle of the mean of the 1971–2010 minus 1931–1970 differences (mm mo^−1^) in CZ50 (blue), CZ100 (cyan), and CZ150 (green).

The change in precipitation between 1931–1970 and 1971–2010 in the CZ varies by season (Fig. 2). CZ50 shows a decrease in precipitation from May to September, CZ100 from July to September, and CZ150 only in August and September (Fig. 4). Positive differences are found in the other months, with the largest value in October. However, the greatest difference by zone occurs in January, where CZ150 has a value of +1.73 (+3.1% increase) and CZ50 only has a difference of +0.38 (+0.5% increase) (Fig. 4). The smallest difference occurs in November. In summary, although climatological precipitation in the CZ reaches a maximum in boreal summer (Fig. 2), the long-term trends reach a maximum in austral summer (Fig. 4).

### Trends in climate normals

**Figure 5:**
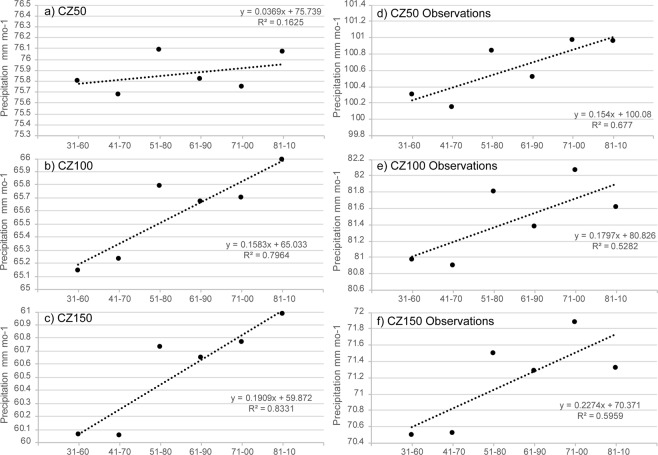
Thirty-year precipitation averages with a 10-year sliding window from 1931–1960 to 1981–2010 of CZ50, CZ100, and CZ150 for the analysis product (**a**–**c**) and observation grid boxes covering the entire 1931–2010 time period (**d**–**f**). Linear trend lines, with equation and r^2^ value, are included in each panel.

Precipitation in the CZ was then averaged over consecutive 30-year reference periods with a 10-year sliding window from 1931–1960 to 1980–2010 following the methodology used in the recent GPCC evaluation study^4^ (see Introduction). Figure 5 shows the time series of the averages for CZ50, CZ100, and CZ150 for the analysis and observation-only products. Overall, the trends are all positive with the best-fit lines having greater slopes for the observation-only data. For CZ50, the climate normals behave similarly between the two products with the exception of 1971–00, where the average is the highest in the observation-only product and second lowest in the analysis product. The 1971–00 reference period continues to be high for the observation-only product for CZ100 and CZ150, whereas for both products the 1931–1960 and 1941–1970 reference periods have substantially lower means than the other climate normals. Also, in both products, precipitation inland experiences a greater upward trend than at the coastline. In the analysis product, a simple linear regression gives a slope of +0.1583 mm mo^−1^ for CZ100. The trend line explains 80% of the variance. For CZ150 the slope is even greater at +0.1909 mm mo^−1^, and the trend line explains 83% of the variance. In both CZ100 and CZ150 the largest outlier reference period is 1951–1980.

**Figure 6:**
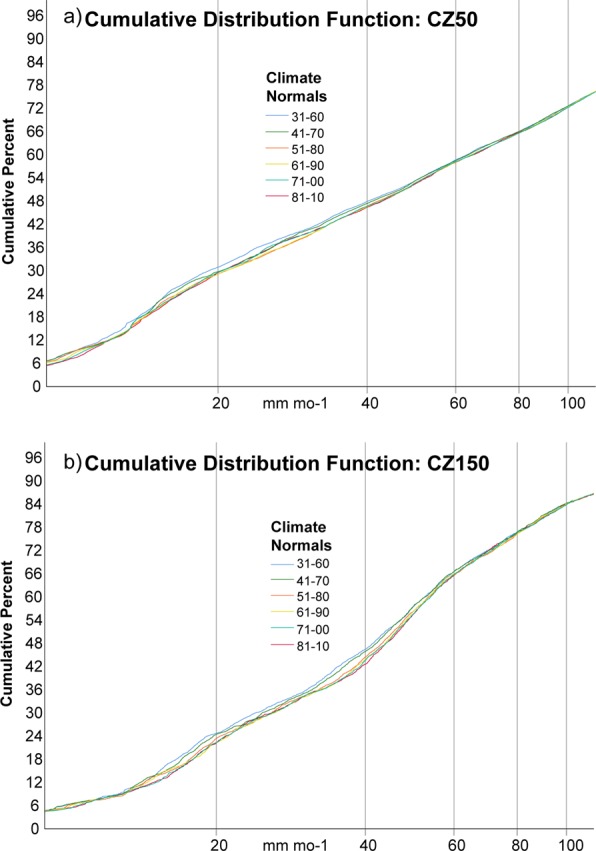
Cumulative distribution functions for (**a**) CZ50 and (**b**) CZ150 of thirty-year precipitation averages (mm mo^−1^) with a 10-year sliding window from 1931–1960 to 1981–2010 (see legend).

While the overall mean precipitation is substantially increasing in the inland bands of the CZ, there is some question as to whether this change is consistent across precipitation rates. Figure 6 compares the cumulative distribution functions (CDF) of the six climate normals derived from the analysis product between CZ50 and CZ150. For both CZ50 and CZ150 the 1931–60 and 1941–70 reference periods were significantly different from the 1981–10 reference period at the 90^th^ percentile, which agrees with Fig. 5. Comparing the shapes of the CDFs, the shoreline has more extreme precipitation, which drives up the overall average, as can be seen from the elongated tail of the CDF as it reaches 100% of the total. Also, percentages increase more rapidly as a function of rain rate near the medians (~40 mm mo^−1^) in the CZ150 CDFs as compared to the CZ50 CDFs. This difference is associated with precipitation at the coastline having a greater frequency of light rain rates and heavy rain rates as compared to further inland (see Fig. 3). The four most recent reference periods all have similar CDFs, which tend to obscure one another in the range of precipitation rates observed in the CZ. However, there is some separation of the CZ150 CDFs (Fig. 6b) near the medians, which is not observed for CZ50 (Fig. 6a). More importantly, the CZ150 CDFs are ordered towards lower percentages chronologically. What this means is that there is a trend towards less light rainfall and more heavy rainfall in the interior of the CZ, which is not observed on the coastline. This is also seen in the trend of median values, which has a monotonic increase over the period of record. For CZ150, the r^2^ of the linear fit of the six medians is 0.92 with a slope of +0.57 mm mo^−1^ per decade. The trend in the median for CZ150 is significant at the 95^th^ percentile assuming a two-tailed Student’s t-test with 3 degrees of freedom.

## Discussion of Potential Mechanisms

A full interpretation of the mechanisms by which the inland CZ would have a greater upward trend in rainfall as opposed to the shoreline would require a globally comprehensive climate analysis with additional high-resolution data sets, and is thus beyond the scope of the current study. However, some speculations are made based on the current literature. First, it is well known that the continents have been warming faster than oceans and this trend is expected to continue^28^. In response to this, studies suggest that the highs in the Northern Hemisphere^29^ and Southern Hemisphere^30^ subtropical oceans are becoming more intense and expanding landward, leading to a greater moisture flux into the CZ. Sea-breeze circulations (SBC) are also dependent on the land-ocean thermal contrast, and the added moisture and energy would be conducive to the generation of mesoscale precipitation fronts that can penetrate further into the interior^31^. Furthermore, if the interior has higher elevations oriented along the coastline, this would result in a greater frequency of orographically enhanced rainfall, boosting the upward trend^11,13^.

**Figure 7:**
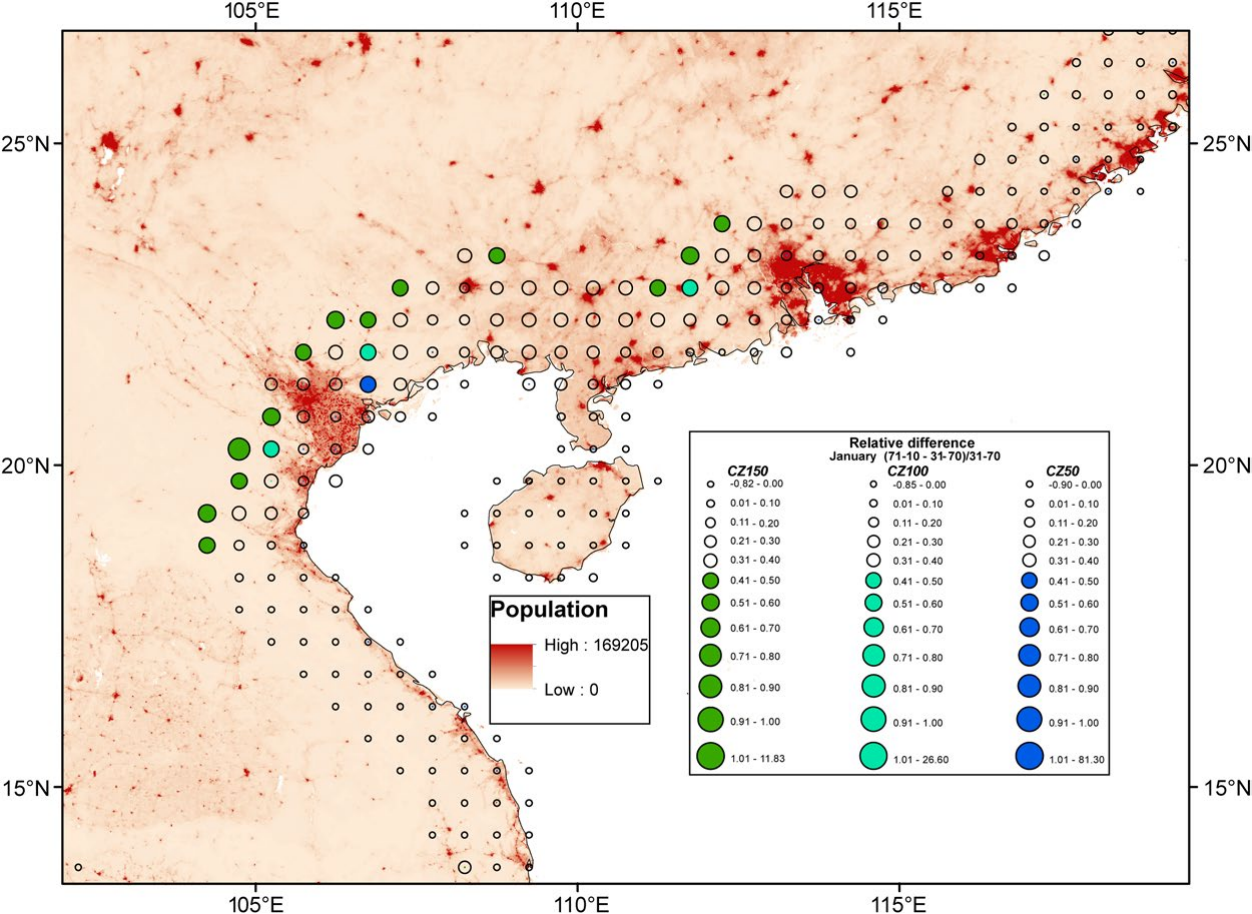
Regional example: Differences in January GPCC V2018 gridded precipitation means between 1971–2010 and 1931–1970 relative to the 1931–1970 period for the coastal zone of southern China. Circles indicate the magnitude of relative differences, with the smallest circles indicating negative differences and color indicating differences greater than 40% (see legend). A population density map is included, where the darker shades of red indicate higher densities. Maps were produced using Desktop ArcMap version 10.5.1 (https://www.esri.com/en-us/home).

Another potential explanation for the trends observed would be urban sprawl in the coastal environment. A future climate simulation for the coastal city of Houston (USA) found that urbanization away from the coast led to a more extensive area of rainfall due to a combination of the SBC and urban heat island (UHI) effect^32^. Another study from São Palo, Brazil found that the UHI acts to increase the propagation speed of the SBC front and advance its position inland beyond the urban area^33^. However, increased urbanization can also lead to a greater concentration of cloud condensation nuclei, which can reduce precipitation locally. Figure 7 shows the 1971–2010 minus 1931–1970 differences in January GPCC analysis precipitation, along with a population density map at the South China Sea coast, where precipitation concentration has been found to be related to urban extent^34^. Precipitation in the interior is increasing faster than at the coast in these rapidly developing metropolitan areas.

Also, it is probable that during the time period studied, cities inland from urbanized coasts became established and developed more rapidly than their coastal counterparts. This can also be seen in Fig. 7 as the areas where the interior has larger relative differences in rainfall compared to the coastline are in the vicinity of “paired” cities in the CZ, namely Hong Kong/Guangzhou and Hai Phong/Hanoi. According to a 3D mesoscale atmospheric model simulation, when two urban centers, one at the coast and one inland, were aligned, the combination of the SBC and UHI led to a chain flow wind pattern, and this wind system penetrated inland faster and lasted longer as compared to the run where only a coastal city existed^35^. Interestingly, the modeled circulation feature became more notable the greater the distance between the two cities (up to 44 km). Finally, the authors’ model results suggested stronger uplift, and thus hypothetically more rainfall, over the inland city compared to the coastal city.

There may be other reasons on the synoptic or planetary scales to explain the results obtained here, but as mentioned in the Introduction, there have been few studies examining the relationship between rainfall and proximity to the coast. It is reasonable to assume that with an increase in atmospheric moisture available in a warming world that extreme rainfall associated with monsoons, oceanic storms (tropical cyclones) and atmospheric rivers, may be extending further inland over the period studied. However, more studies and additional precipitation data, including individual gauge observations in the CZ, are needed to verify the results presented here and inform the mechanisms driving the trend towards inland areas of the CZ becoming wetter faster.

## Conclusions

Climatologies of global terrestrial and oceanic precipitation, and their trends, have been estimated a number of times using several global precipitation data sets for the primary purpose of evaluating the hydrologic cycle, yet few attempts have been made to construct a long-term climatology of global precipitation at the land-sea interface. However, low-lying coastal areas are flood prone and contain a large proportion of the world’s population. Also, global climate models do not capture the spatial patterns and timing of rainfall at the coast^36^. This study divided the coastal zone into three domains and examined precipitation means, distributions, and trends using a state-of-the-art global high-resolution gridded data set.

As expected, the 1931–2010 mean and annual cycle of precipitation near the coastline contains features of previously derived oceanic climatologies, while the inland domains are more similar to terrestrial climatologies. There is a pronounced decrease in mean rainfall from the coast inland, and while rainfall throughout the coastal zone peaks in the boreal summer, precipitation remains high on the coastline into the fall, which may be related to Northern Hemisphere tropical cyclones.

A separate examination of the first and second halves of the record revealed that precipitation is increasing in the coastal zone in the October to April time period. Interestingly, the positive trend in precipitation is more pronounced over the two inland domains as compared to the coastline in each of these months, except November. Between 100 and 150 km of the ocean rainfall increased 10.2 mm yr^−1^ (0.9 mm mo^−1^) or 1.4%, whereas within 50 km of the coast, rainfall increased by only 2.4 mm yr^−1^ (0.2 mm mo^−1^) or 0.3%. An analysis of the distribution of the precipitation differences across the two time periods revealed significant differences among the three spatial domains.

Precipitation in the coastal zone was averaged over six climate normals from 1931–1960 to 1981–2010. The coastline population showed a weak positive trend compared to the domains from 50 to 100 km away from the coastline and 100 to 150 km away from the coastline. An examination of the cumulative distribution functions revealed that the trends from 1931–1960 to 1981–2010 in the most inland zone are most pronounced for rain rates near the median ~45 mm mo^−1^. In fact, the trend in the median rain rate is significant at the 95^th^ percentile.

Finally, mechanisms for the gradient in the precipitation trends within the coastal zone were proposed, which included an enhancement of the sea-breeze circulation and urban heat island effect, and the potential for rain systems to bear more rainfall further inland. While global sea level rise is a well-known consequence of climate change, this is the first study to add the uneven increase of rainfall as a climate problem that is likely to be confronted by ecosystems and society in the coastal zone. The rainfall generated in the interior of the coastal zone will eventually make its way to the sea though river systems, exacerbating future coastal flooding beyond current expectations based on oceanic changes alone (sea-level rise, storm surge, waves, and tides)^37^. One limitation of the current study is the spatial resolution of the GPCC data. GPCC also produces a 0.25° product which could be considered in future studies, but since there is no difference in the number of gauge observations, it is expected that the results (especially the observation-only results) will remain unchanged. However, additional studies and complementary data sets are encouraged to better understand how the coastal zone may be transformed by excess water from both the ocean and the atmosphere in a warming world.

## Data and Methods

This study uses the monthly Global Precipitation Climatology Centre (GPCC) full data reanalysis version 2018, which interpolates rain gauge data onto a 0.5° latitude/longitude resolution grid^38^. The data set extends from January 1901 to December 2013. Gauge density used to construct GPCC varies over time, but is based on over 75,000 stations with climatological normals^4^. GPCC has more stations than any other gauge-based precipitation product and undergoes a thorough quality control. The peak in gauge density occurs from 1931 to 2010, and therefore this time period was chosen for further analysis^3^ (see their Fig. 2). GPCC’s procedure is here summarized. Anomalies from the climatological normals are interpolated at the stations to a 0.5° mesh grid. The interpolation is a modified version of SPHEREMAP^39,40^. Next, an average is taken at the four corners of the grid to produce an areal average. The areal average is weighted by the land fraction. Finally, the gridded anomalies are added to the background climatology. The sampling error is reduced by interpolating the anomalies, instead of the absolute values, and is deemed more beneficial than the type of interpolation technique. An observation-only product was also used in this study where precipitation is only considered for grid cells that contain at least one gauge observation (see Fig. S1). The people per pixel 2015 population density data was taken from WorldPop (www.worldpop.org.uk/data/data_sources).

After annual and monthly climatologies of precipitation were computed, the gridded data was imported into ArcMAP 10.5.1 as geotiff images and then converted to points. Antarctica is not included in this analysis. Two world continent shapefiles, originally downloaded from Environmental Systems Research, Inc., were added: one as a polygon and one as a line feature. The projection is WGS84 with a 1:15000000 scaling. The layers are part of the 2014 ESRI Data and Maps collection for ArcGIS 10.2. 50 km, 100 km, and 150 km buffers were created around the “coastlines” of the continents. CZ50 was constructed by selecting all GPCC points contained within the 50 km buffer. CZ100 (CZ150) was constructed by selecting all GPCC points contained within the 100 km (150 km) buffer, and then deselecting the points that were outside the continent polygon.

The analysis consists of simple descriptive and nonparametric statistics. A Kolmogorov-Smirnov test was used to test the null hypothesis that the distribution of 1971–2010 minus 1931–1970 precipitation differences is the same between CZ50 and CZ150, and an independent samples Kruskal-Wallis test was used to test the null hypothesis that the CDFs of precipitation were the same between climate normals.

## Supplementary information

Supplementary Figure S1

## Data Availability

The datasets generated during and/or analysed during the current study are available from the corresponding author on reasonable request.
