# Supplementary material for: Means and Long-Term Trends of Global Coastal Zone Precipitation
Source: Sci Rep. 2019 Apr 1;9:5401. doi: 10.1038/s41598-019-41878-8 (PMC6443665; doi:10.1038/s41598-019-41878-8)
Supplement: Supplementary file 1 — Supplementary Figure S1 [file 41598_2019_41878_MOESM1_ESM.docx]

*Scientific Reports*

Supporting Information for

Means and Long-Term Trends of Global Coastal Zone Precipitation

Scott Curtis

Distinguished Professor in Natural Sciences and Mathematics

Department of Geography, Planning, and Environment

East Carolina University, Greenville, NC 27858, USA

curtisw@ecu.edu

**Contents of this file**

- One figure: S1


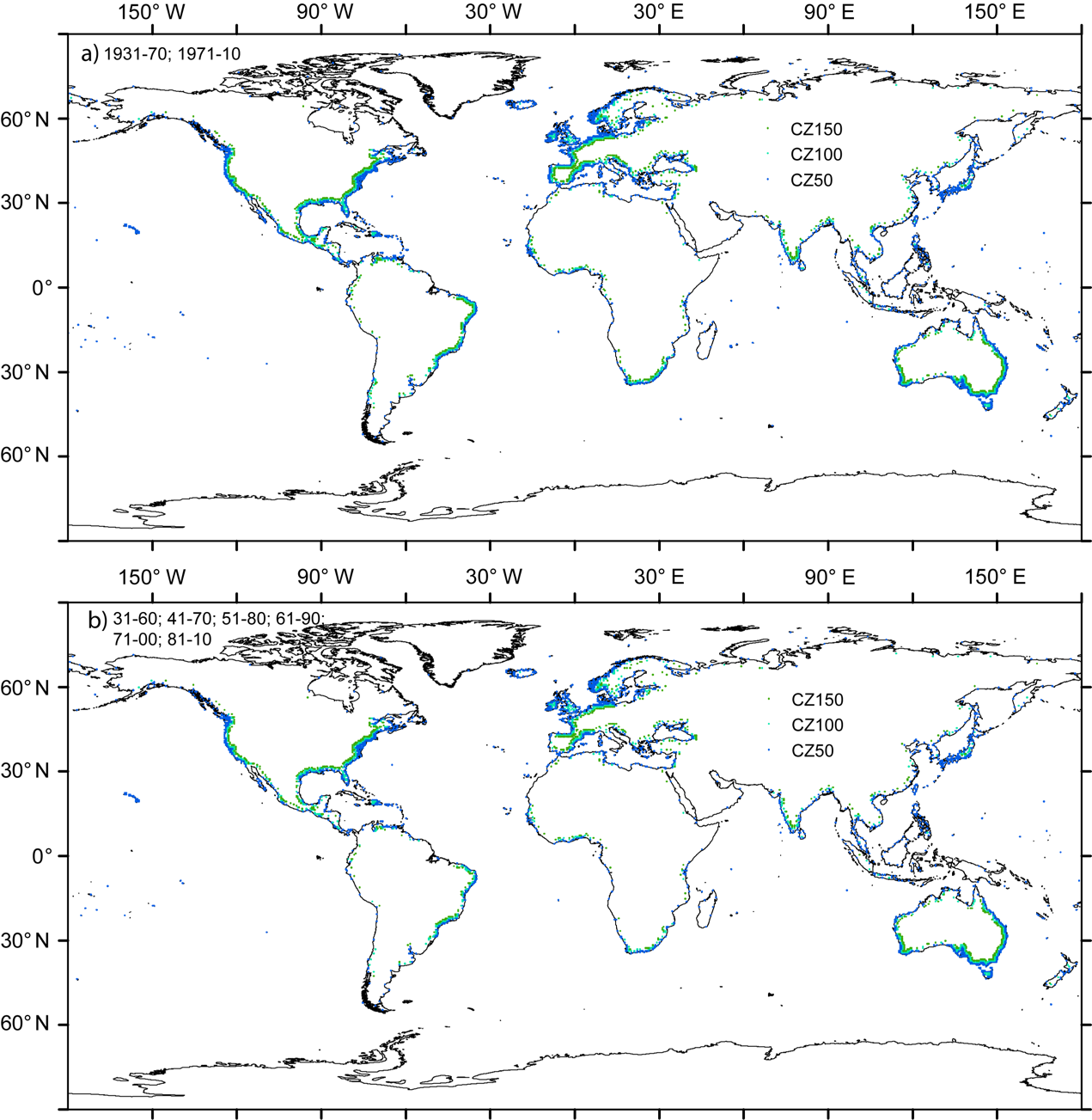


Figure S1. Grid boxes from the observation-only GPCC V2018 product used to analyze precipitation within CZ50, CZ100, and CZ150. a) grid boxes with observations in both the 1931-1970 and 1971-2010 time periods and b) grid boxes with observations in the 1931-60, 1941-70, 1951-80, 1961-90, 1971-00, and 1981-10 reference periods.
